# Heterogeneity of Systemic Oxidative Stress Profiles in COPD: A Potential Role of Gender

**DOI:** 10.1155/2015/201843

**Published:** 2015-06-17

**Authors:** Jonathan Maury, Farés Gouzi, Philippe De Rigal, Nelly Heraud, Joël Pincemail, Nicolas Molinari, Pascal Pomiès, Dalila Laoudj-Chenivesse, Jacques Mercier, Christian Préfaut, Maurice Hayot

**Affiliations:** ^1^PhyMedExp, University of Montpellier, INSERM U1046, CNRS UMR 9214, 34295 Montpellier Cedex 5, France; ^2^University of Montpellier, 34295 Montpellier, France; ^3^Clinique du Souffle “La Solane”, Fontalvie Group, 66340 Osséja, France; ^4^Department of Clinical Physiology, CHRU Montpellier, 34295 Montpellier, France; ^5^Department of Cardiovascular Surgery and CREDEC, University of Liège, CHU Sart Tilman, 4000 Liège, Belgium

## Abstract

Oxidative stress (OS) plays a key role in the muscle impairment and exercise capacity of COPD patients. However, the literature reveals that systemic OS markers show great heterogeneity, which may hinder the prescription of effective antioxidant supplementation. This study therefore aimed to identify OS markers imbalance of COPD patients, relative to validated normal reference values, and to investigate the possibility of systemic OS profiles. We measured systemic enzymatic/nonenzymatic antioxidant and lipid peroxidation (LP) levels in 54 stable COPD patients referred for a rehabilitation program. The main systemic antioxidant deficits in these patients concerned vitamins and trace elements. Fully 89% of the COPD patients showed a systemic antioxidant imbalance which may have caused the elevated systemic LP levels in 69% of them. Interestingly, two patient profiles (clusters 3 and 4) had a more elevated increase in LP combined with increased copper and/or decreased vitamin C, GSH, and GPx. Further analysis revealed that the systemic LP level was higher in COPD women and associated with exercise capacity. Our present data therefore support future supplementations with antioxidant vitamins and trace elements to improve exercise capacity, but COPD patients will probably show different positive responses.

## 1. Introduction

Chronic obstructive pulmonary disease (COPD) is a complex disease usually characterized by progressive airflow limitation that is not fully reversible and significant extrapulmonary effects that may further contribute to disease severity in individual patients [1]. One of the main systemic effects is a decrease in muscle mass linked to muscle dysfunction, which contribute to the decline in exercise capacity and a worsened prognosis [2, 3]. Although many factors are implicated in the respiratory and muscle pathophysiology of COPD, oxidative stress (OS) appears to play a key role [4, 5]. The COPD literature usually describes an increase in prooxidants, macromolecular damage (lipid and protein oxidation), and DNA oxidation [6–8], which correspond to deleterious OS as defined by Jones [9]. To limit cell damage, a complex antioxidant system may directly scavenge ROS and/or inhibit lipid peroxide reactions [10–13], but previous studies have shown a decrease in many enzymatic and nonenzymatic antioxidants in COPD patients [6, 7, 14–16].

However, the literature also suggests that systemic OS markers show great heterogeneity, particularly in the systemic antioxidant levels. For example, for a given parameter, systemic antioxidant levels in different groups of COPD patients were either lower than [6, 7, 14] or equal to [5] the levels in healthy subjects. The discrepancies among studies may be due to the differences in centers and the low number of COPD patients included in the investigations. The literature has also described great heterogeneity from one COPD patient to another suggesting different systemic OS marker profiles, but none of these earlier studies has tested this hypothesis [5–8, 14]. The impact of such clinical factors as physical inactivity, tobacco consumption, gender, or nutritional intake on prooxidants and antioxidant levels may explain the individual differences in systemic OS markers among COPD patients but the literature remains unclear [17–19]. Similarly, although it is broadly acknowledged that deleterious OS is implicated in muscle pathophysiology [5], only one study showed that the level of systemic isoprostanes, a specific marker of lipid peroxidation, was more elevated in a COPD phenotype characterized by muscle atrophy and decreased exercise capacity [8].

A more systematic analysis of antioxidant deficits and deleterious OS markers in COPD patients is thus needed to understand the great heterogeneity in the results reported in the literature and to provide data that can better guide the prescription of antioxidant supplementations. Therefore, using validated and previously published reference values determined from a cohort of healthy subjects [20, 21], this study aimed to identify OS marker imbalances in COPD patients and to determine whether systemic OS profiles exist. The secondary objective was to identify the clinical and muscle characteristics specifically associated with these systemic OS markers in COPD patients.

## 2. Materials and Methods

### 2.1. Study Patients

Fifty-four stable COPD patients, as defined by the Global Initiative for Chronic Obstructive Lung Disease (GOLD) guidelines, were included in our study with the diagnosis confirmed by plethysmography (Body Box  5500, Medisoft, Belgium). The parameters evaluated during plethysmography were compared with normal values [22] and the diagnosis of COPD was especially based on a postbronchodilator forced expiratory volume in one second (FEV_1_)/forced vital capacity (FVC) ratio below 70% of theoretical FEV_1_/FVC [1]. Exclusion criteria were the presence of exacerbations within the last month, unstabilized disease (e.g., cardiac, inflammatory, and neuromuscular), disability that could modulate OS and limit exercise capacity, antioxidant supplementation (vitamins, trace elements, etc.), and use of drugs such as allopurinol and N-acetylcysteine within the last month or use of oral corticosteroids over the last six months. All had been referred for a rehabilitation program at “La Solane” Pulmonary Rehabilitation Center, in Osséja, France. All patients received a detailed information letter about the study before providing their written informed consent. This study was approved by the Ethics Committee Montpellier Sud-Méditerranée IV (n°2011-A00842-39) and conducted in accordance with the Declaration of Helsinki and the European guidelines for “good clinical practice.”

### 2.2. Oxidative Stress Determination

Venous blood samples were taken immediately after an overnight fast and drawn into tubes containing EDTA or Na-heparin as anticoagulant or clot-activating gel, depending on the parameter. Blood samples were immediately conditioned in the laboratory of “La Solane” Pulmonary Rehabilitation Centre, Osséja, France. Firstly, the samples from one ethylenediaminetetraacetic acid (EDTA) tube were directly transferred to Eppendorf tubes to analyse systemic levels of enzymatic antioxidants: total reduced glutathione (GSH = 50 *μ*L), oxidised glutathione (GSSG = 100 *μ*L), superoxide dismutase (SOD = 500 *μ*L), and peroxidase glutathione (GPx = 500 *μ*L). For the GSSG sample, we added 10 *μ*L of scavenger (Bioxytech GSH-GSSG 2 mL) to keep GSSG in its oxidised form. For SOD, we washed the blood sample with distilled water and centrifuged it at 3500 T/min for 5 minutes. We repeated this process four times and to finish we collected 500 *μ*L of plasma conditioned with 500 *μ*L of demineralised water. Secondly, other blood samples were collected in ethylenediaminetetraacetic acid (EDTA) and sodium and lithium heparin tubes and were immediately centrifuged (3000 T/min for 10 min). Then, plasmas were frozen in dry ice and kept at −80°C until analysis of lipid peroxidation markers, vitamins, and trace elements. Specifically, for vitamin C levels, 500 *μ*L of plasma was immediately transferred to ice-cold tubes containing 500 *μ*L of 10% metaphosphoric acid (to protect against vitamin C oxidation with air) and then frozen in dry ice. Within a maximal period of one month, samples were sent for analysis in dry ice to the CHU Clinical Laboratories of the University of Liège-Belgium (Professor Joël Pincemail). For each OS marker, this laboratory established lower and upper limits of the reference values (LLR and ULR) from a large healthy subject cohort, as previously done [20, 21]. This cohort included 123 subjects of Caucasian origin with an age range of 21 to 64 years with no known history of respiratory, cardiovascular, liver, or kidney diseases or cancer.

#### 2.2.1. Lipid Peroxidation Markers

The lipid peroxides (ROOH) in plasma were analyzed with a commercial kit (Oxystat, Biomedica Gruppe, Vienna, Austria) as previously described [21, 23]. Oxidized low-density lipoprotein (LDL) levels in plasma samples were determined spectrophotometrically with a competitive enzyme-linked immunosorbent assay (ELISA) kit (Immunodiagnostik, Bensheim, Germany).

#### 2.2.2. Antioxidants

For vitamin C determination, 0.5 mL plasma was immediately transferred to ice-cold tubes containing 0.5 mL of 10% metaphosphoric acid and kept at −80°C. Analyses were performed by a spectrophotometric method using the reduction of 2,6-dichlorophenolindophenol (Perkin Elmer Lambda 40, Norwalk, CT, USA). Plasma vitamin A and vitamin E (*α*- and *γ*-tocopherol) levels were determined simultaneously by HPLC (Alliance HPLC System, Waters Corp., Milford, MA, USA) coupled with a diode array detector [24]. Blood levels of vitamin E were normalized to plasma levels of total cholesterol [25], which were determined by an enzymatic method with cholesterol oxidase. Total reduced glutathione (GSH) and oxidized glutathione (GSSG) were determined in whole blood using the GSH/GSSG-412 kit (Bioxytech, Oxis International, Inc., Portland, WA, USA). Superoxide dismutase (SOD) and peroxidase glutathione (GPx) enzymatic activities in whole blood were determined with the Ransod and Ransel kits (Randox, England) and were expressed as UI/g of haemoglobin.

#### 2.2.3. Trace Elements

Plasma levels of selenium, copper, and zinc were determined by inductively coupled plasma-mass spectroscopy [26].

### 2.3. Functional Evaluations

*Bioelectrical multifrequency impedance* (BIACORPUS RX spectral, MEDICAL HealthCare GmbH, Karlsruhe, Germany) was used to assess body composition: fat mass (FM), fat-free mass (FFM), total body water, and muscle mass. More specifically, muscle mass was calculated using the bioelectrical impedance analysis equation of Janssen and colleagues [27].

*A maximal incremental exercise test* on a cycle ergometer (Ergoselect, Sorinnes, Belgium) was performed to evaluate exercise capacity. The 3-minute warm-up was performed at 20% of maximal predicted power, and the workload was increased every minute by 8% of maximal predicted power. Recording with a 12-lead electrocardiogram and gas exchange measurement with a breath-by-breath system (Medisoft Expair software, Ergocard, Sorinnes, Belgium) were continuous during exercise testing. The maximality criteria were established in accordance with the international standards on cardiopulmonary exercise testing [28]. Maximal power output (W_sl_) and symptom-limited oxygen consumption (VO_2_sl) were the main variables evaluated.

*The 6-minute walk test (6-MWT)* [29] was also performed to evaluate exercise capacity. It was performed twice along a 30 m perimeter with at least 30 minutes between tests and the highest 6-minute walking distance (6-MWD) was selected, in line with the guidelines [29]. Oxygen saturation (SpO_2_) and heart rate (HR) were recorded every minute using pulse oximetry (Nonin 8500 M, Nonin Medical, Inc., Minneapolis, MN, USA). The dyspnea score was measured before and at the end of the test on a visual analog scale.

*The BODE index* was used to assess global disease severity [30]. This index is based on the body mass index (B), the degree of airflow obstruction with FEV_1_ (O), dyspnea (D) assessed by the Medical Research Council (MRC) scale, and exercise capacity (E) measured by the 6-MWT.

*The quadriceps maximal voluntary isometric contraction* (QMVC, in Nm) was measured in a seated position at 90° knee and hip flexion on a quadriceps-hamstring chair (Quadriergoforme Rehabilitation Chair, Aleo Industrie/Design Corporel, Salome, France). This chair has a strain gauge system connected to a signal acquisition and analysis system (MP36, BIOPAC Systems). Three reproducible measurements (within 5%) per leg were recorded and the best value of the dominant leg was retained. QMVC was compared with the reference values [2].

*Quadriceps endurance time* (Qend, in seconds) was determined only for the dominant leg, as previously described by our group [8]. The patients performed knee extensions (6 movements per minute) with a workload to 30% of QMVC until exhaustion. Immediately after this test, patients performed a QMVC to evaluate quadriceps fatigue, and a reduction of QMVC >10% was necessary to validate the test.

*The questionnaire validated by Voorrips et al.* was used to assess the physical activity level [31]. This questionnaire is composed of three parts, with information about daily activities, sports, and spare-time activities giving a total score. The daily physical activity value is interpreted as low (total score < 9.4), moderate (total score between 9.4 and 16.4), or high (total score > 16.4).

### 2.4. Nutritional Assessment

The nutritional status of all patients was obtained from dietary records [32] that they maintained in the week before the start of the functional evaluations. We then determined the potential origins of nonenzymatic antioxidant deficits. Patients recorded on paper the foods and beverages consumed over three nonconsecutive days, specifying quantities based on various indicators (weighing, egg cups, tablespoons, food models, etc.). With the collaboration of a dietician to determine the volume of food intake for each patient, nutrients were converted to micronutrients in order to estimate the consumption of antioxidants like vitamins and trace elements using GENI software (Micro6, Villers-lès-Nancy, France). This software compares an individual's dietary intake with a recommended daily intake (RDI) given by the “Agence Nationale de Sécurité Sanitaire de l'Alimentation, de l'Environnement et du Travail (ANSES)” of France. These RDIs are based on gender and age [33].

### 2.5. Statistical Analysis

Quantitative data are presented as mean ± standard deviation (SD). Individual values for each OS marker were compared with LLR and ULR from the healthy subject cohort in order to obtain the percentage of COPD patients with abnormal values (below LLR and above ULR). Using principal component analysis (PCA), we determined new variables (major components) summarizing the multiple systemic OS markers measured. Then, each variable and each patient were placed on the graph/map of PCA. Using Ward's method, we performed cluster analysis based on the major components of PCA. A one-way analysis of variance (or the Kruskal-Wallis for nonnormally distributed variables) or Pearson's chi-squared test compared the OS and clinical parameters of the identified COPD patient clusters. If interactions between clusters were found, post hoc analysis (Bonferroni or Dunn) was performed to identify specific differences. A Student's *t*-test or a Mann-Whitney* U* test was used to compare the clusters separately. The Pearson coefficient (univariate analysis) identified the clinical and/or functional parameters associated with changes in the OS markers. Then, a multivariate analysis (ANCOVA) was performed to determine the factors independently associated with the OS markers, particularly with systemic lipid peroxidation. We chose this specific OS marker because it is strongly implicated in the cell damage induced by redox imbalance. In this multivariate analysis model, we studied the factors that might be associated with and/or modify the systemic lipid peroxidation level, such as age, disease severity (FEV_1_ and BODE score), physical activity (Voorrips score), gender, and tobacco consumption. Also, we included in this model the parameters significantly correlated with systemic lipid peroxidation, like 6-minute walking distance (6-MWD) and muscle mass index.

For all analyses, the level of significance was set at *p* < 0.05. Statistical analyses were performed using R version 2.15.2.

## 3. Results

The anthropometric and clinical characteristics of the 54 COPD patients are presented in Table 1. An equivalent number of men and women were included (27 men and 27 women; mean age 60 ± 7 years with an age range of 40 to 80 years) and all GOLD severity stages (GOLD I to GOLD IV) are represented.

### 3.1. Systemic OS Imbalance in COPD Patients

**Figure 1:**
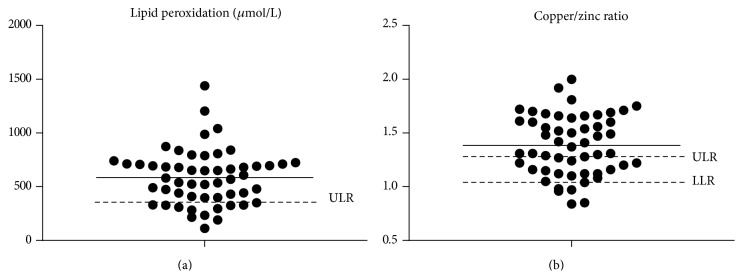
Systemic lipid peroxidation level and copper/zinc ratio in 54 COPD patients. Closed circles: individual values. Lower dashed line: lower limit of reference (LLR) [22]. Upper dashed lines: upper limit of reference (ULR) [22]. Solid line: mean value of the COPD patient group.

**Figure 2:**
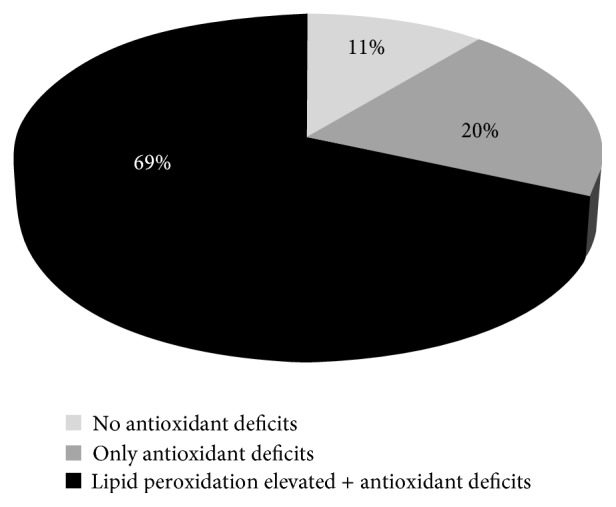
Prevalence of patients with plasma antioxidant deficits and elevated lipid peroxidation in 54 COPD patients. Black: patients with elevated plasma lipid peroxidation and at least one antioxidant deficit/redox imbalance. Gray: patients with only antioxidant deficit/redox imbalance. White: patients with a normal systemic oxidative stress profile.

The most prevalence of COPD patients with deficits (values below the LLR) in nonenzymatic antioxidants was found in vitamin C, zinc, and selenium (Table 2). These deficits contributed to an imbalance in the vitamin C/vitamin E and copper/zinc ratios (Table 2). Although less than 10% of the COPD patients had systemic copper deficits, 61% had an elevated copper/zinc ratio (Table 2). The systemic copper/zinc ratio and the copper levels were significantly and positively correlated with the systemic lipid peroxidation levels (*p* < 0.001; *r* = 0.69 and *p* < 0.001; *r* = 0.83, resp.). Our results showed that 69% and 30% of COPD patients had systemic lipid peroxidation levels and LDL oxidized above the ULR, respectively (Table 2). In addition, it should be noted that despite the great variability in values for each parameter as illustrated in Figure 1, 89% of the COPD patients had at least one antioxidant deficit (Figure 2).

**Figure 3:**
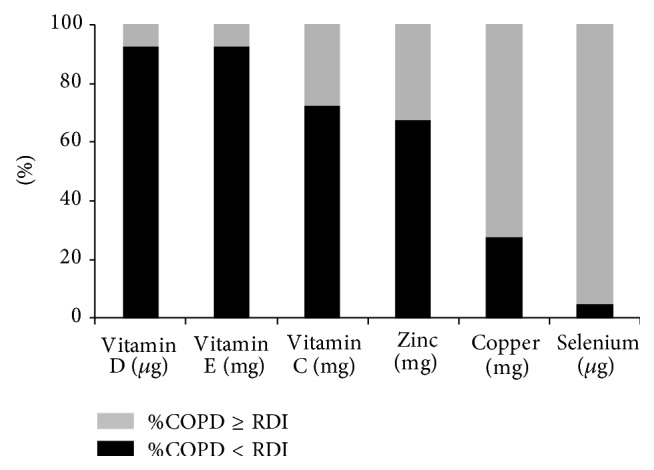
Quality of micronutrient intake in 40 COPD patients. For each micronutrient, results showed the proportion of patients with COPD with dietary intake above (grey part) or below (dark part) recommendations. Recommended daily intake (RDI) was defined in function of gender.

Our data also revealed a substantial imbalance in dietary intake, in particular with regard to micronutrients, although we observed no significant correlation with systemic antioxidant levels. Intake of vitamin C and E was below the RDI in 70% and 92% of COPD patients, respectively (Figure 3). Regarding trace element consumption, absolute intake was below the RDI for zinc and copper in 68% and 27% of COPD patients, respectively, while selenium intake was normal (Figure 3).

### 3.2. Profiles of Systemic OS Markers in COPD Patients

**Figure 4:**
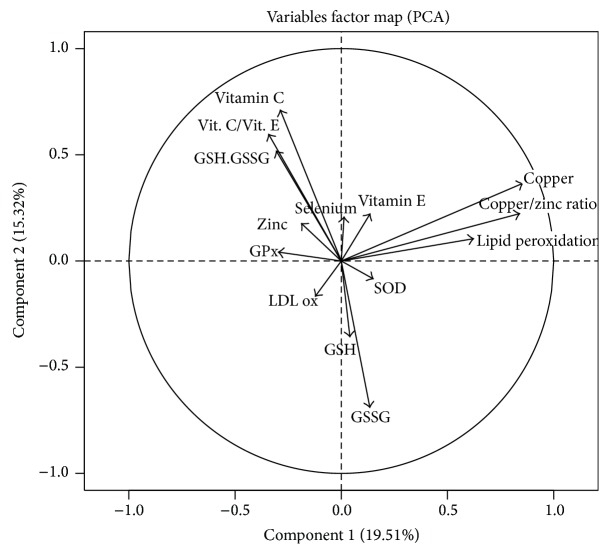
Factor map of 14 systemic OS markers obtained in all individuals (*n* = 54) by principal component analysis (PCA). The original variables are projected in a reduced dimension space defined by component 1 (*x*-axis) and component 2 (*y*-axis).

PCA was performed on 14 systemic OS markers, which yielded two major components (Figure 4). Component 1 was defined essentially by systemic copper, the copper/zinc ratio, and lipid peroxidation levels, whereas component 2 was defined by systemic GSH, GSSG, GSH/GSSG, vitamin C, and the vitamin C/vitamin E ratio levels. Four clusters of COPD patients emerged from the hierarchical model (Ward's method) of classification in function of the 2 major components defined in PCA.

Cluster 1 comprised COPD patients with no specific OS markers deficits in common. Cluster 2 comprised COPD patients with a more elevated systemic lipid peroxidation level than that of cluster 1 patients, whereas the systemic GSH/GSSG ratio was lower (Table 3). Compared with clusters 1 and 2, the COPD patients of clusters 3 and 4 had significantly higher levels of systemic lipid peroxidation and copper and significantly higher copper/zinc ratios (Table 3). More specifically, cluster 4 had significantly lower levels of systemic vitamin C (and vitamin C/vitamin E ratio), GSH, and GPx compared with cluster 3, while the GSH/GSSG ratio remained normal (Table 3).

### 3.3. Clinical Relevance of the Systemic OS Imbalance

Clinical and muscle parameters of the four clusters of COPD patients are presented in Table 4. Significant differences were found in the sex ratio and muscle mass index. Compared with clusters 1 and 2, clusters 3 and 4 included significantly more women (Table 4).

**Figure 5:**
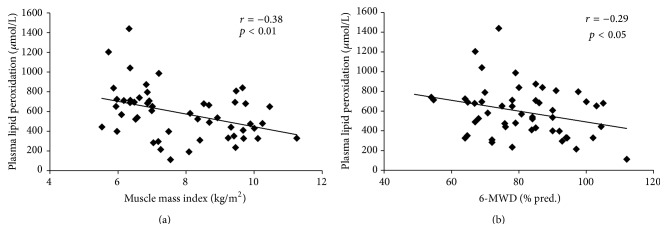
Relationships between systemic lipid peroxidation and (a) muscle mass index (kg/m^2^) and (b) 6-MWD (% pred.). (a) Systemic lipid peroxidation, expressed in *μ*mol/L, was significantly and negatively correlated with muscle mass index (*r* = −0.38; *p* < 0.01) in 54 COPD patients. (b) Systemic lipid peroxidation, expressed in *μ*mol/L, was significantly and negatively correlated with 6-MWD (*r* = 0.29; *p* < 0.05) in 54 COPD patients.

When the systemic OS markers were considered separately, univariate analysis revealed a significant and negative correlation between the copper/zinc ratio and the muscle mass index (*p* < 0.05; *r* = −0.49). Similarly, systemic lipid peroxidation was significantly and negatively correlated with the muscle mass index (*p* < 0.01; *r* = −0.38; Figure 5(a)) and the 6-MWD in % predicted (*p* < 0.05; *r* = −0.29; Figure 5(b)).

**Figure 6:**
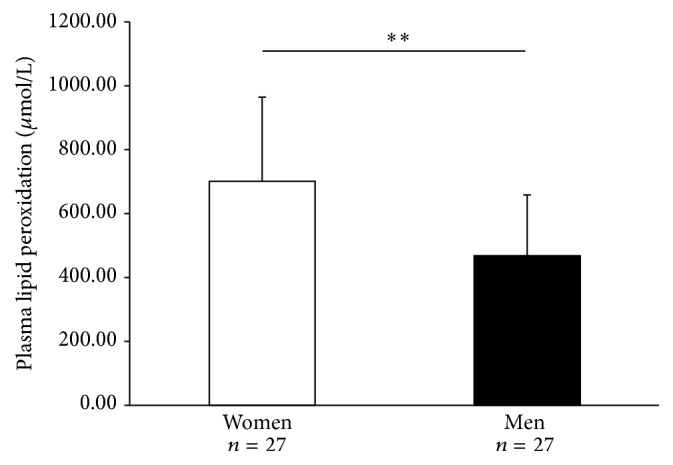
Systemic lipid peroxidation levels according to gender in COPD patients. Bars represent results expressed in mean ± standard deviation. White bars: results of 27 COPD women. Black bars: results of 27 COPD men. ^*∗∗*^ *p* < 0.01.

The multivariate analysis model also showed that systemic lipid peroxidation was associated with gender (Table 5). As shown in Figure 6, the mean value of systemic lipid peroxidation was significantly higher in COPD women than in COPD men (701 ± 263 *μ*mol/L versus 468 ± 190 *μ*mol/L; *p* < 0.01). Secondly, systemic lipid peroxidation was significantly associated with the 6-MWD (% predicted) (Table 5). Moreover, the negative correlation between systemic lipid peroxidation and the 6-MWD (in % predicted) was significant only in women (*p* < 0.05; *r* = −0.49).

## 4. Discussion

This study showed that stable COPD patients referred for a rehabilitation program had a systemic antioxidant imbalance, particularly regarding vitamins and trace elements. Taken together, 89% of COPD patients had at least one systemic antioxidant imbalance, which might explain the elevated systemic LP levels found in 69% of them. We confirmed the great heterogeneity of systemic OS markers in these patients, but for the first time we also identified four profiles of systemic OS marker imbalance. The profiles of the COPD patients of clusters 3 and 4 were characterized by a considerable imbalance in copper metabolism, and cluster 4 patients showed imbalances in GSH, GPx, and vitamin C associated with a particularly high level of systemic lipid peroxidation. Regarding the clinical and muscle parameters, further analysis allowed us to identify the fact that the systemic lipid peroxidation level was higher in COPD women and was associated with a decrease in exercise capacity.

### 4.1. Systemic Antioxidant OS Profiles in COPD Patients

Our results showed specific OS markers imbalance, in particular in nonenzymatic antioxidants, in different proportions, but 89% of COPD patients were concerned. Some OS markers showed a great part of deficits in COPD patients, as vitamin C (26%), zinc (28%), and selenium (66%) [15], while other markers as GSH, vitamin E, and enzymatic antioxidants are not much affected compared with that described in the literature [7, 14, 15, 34]. These discrepancies between studies cannot be linked only to a small number of COPD patients included or methodological differences. In fact, to limit these methodological considerations, we compared systemic OS markers of COPD patients to validated reference values obtained in a large cohort of healthy subjects [20, 21]. One possible limitation of this methodology is the different range of our COPD patients included (47 to 76 years) versus this cohort of healthy subjects (21 to 64 years) [20, 21]. Indeed, in the present study, only 28% of patients were aged 65 years or more. However, the effect of age on systemic OS markers in particular in antioxidants was discussed and questioned in literature [35, 36]. In agreement with this discussion, no difference was noted for any of the systemic OS markers investigated in our study between COPD patients 65 years or younger (47 to 64 years) and COPD patients older than 65 years (data not shown). Despite the use of this methodology which appeared as a suitable solution, the apparent discrepancies between studies reflect a large heterogeneity in individual systemic OS marker in COPD patients as illustrated in our study (Figure 1).

One of the most important factors that could modulate and explain systemic nonenzymatic antioxidant differences between COPD patients is the nutritional intake [37, 38]. However, we did not find any correlation between micronutrient intake and systemic antioxidant levels although our study showed a high prevalence of inadequate intake of micronutrients like vitamins C, E, and zinc in COPD patients, as shown by Van de Bool et al. [39]. These data suggest specific underlying mechanisms for antioxidant system imbalance as reflected by the cluster analysis results. Indeed, we showed that clusters 3 and 4 had elevated systemic copper level and copper/zinc ratio. In addition, we found a significant correlation between systemic lipid peroxidation and copper (and copper/zinc ratio) in line with previous studies [21, 40]. In literature, it is well admitted that copper in excess may have a “prooxidant” activity inducing lipid peroxidation and zinc may partially inhibit these reactions [41]. As nutritional intake did not appear to explain the increase in systemic copper in our study, an inflammatory phenomenon and/or a more important susceptibility to tobacco consumption are currently the only assumptions advanced in literature [42, 43].

Compared with clusters 2 and 3, the COPD patients of cluster 4 had normal values for the GSH/GSSG ratio, whereas the mean lipid peroxidation level was more elevated. However, in the deleterious OS state, the GSH/GSSG ratio was usually decreased, reflecting the activity of GSH to scavenge ROS, thereby inducing an increase in this oxidized form (GSSG) [12]. To explain this surprising result, we hypothesize that GSH is not used by glutathione peroxidase (GPx). In fact, GSH acts mainly as a substrate of GPx, which directly scavenges ROS and may limit lipid peroxidation production [11]. It was shown that GPx activity will respond in two different ways to oxidative stress: the first is an adaptation characterized by an increase in GPx activity, whereas, in response to chronic or severe oxidative stress, this activity decreases [2, 3]. We were able to associate this GPx kinetics with the results in our four clusters of COPD patients. The first three clusters had elevated GPx levels, whereas cluster 4 COPD patients showed a tendency towards a decrease in systemic GPx. Nevertheless, the current data in literature did not allow us to check this hypothesis.

Moreover, as they act as substrate, the decrease in selenium and zinc in a large part of COPD patients may also contribute to the decrease in the enzymatic activity of GPx and SOD, respectively [10, 11]. At last, this decrease in GSH activity may contribute to the decrease in vitamin C observed in cluster 4. In fact, GSH regenerates the oxidized form of vitamin C in its active form [13]. Also, vitamin C directly scavenges ROS and permits the oxidized form of vitamin E to be regenerated to maintain normal systemic levels as found in our study [13].

### 4.2. Clinical Relevance of Systemic OS Imbalance

It is widely acknowledged that antioxidant deficits might contribute to deleterious OS, as illustrated by the significant and positive correlation found between systemic copper/zinc ratio and lipid peroxidation [44]. In agreement with previous studies [8, 14], we found an increase in systemic lipid peroxidation in 69% of COPD patients. The different antioxidant imbalance profiles described previously may explain higher systemic lipid peroxidation levels found in clusters 3 and 4 of COPD patients. Interestingly, we also observed that clusters 3 and 4 of COPD patients were constituted mainly of women. The literature had usually described that men generated lower levels of enzymatic antioxidant, potentially leading to higher OS [45]. However recent studies argued that women have a more important OS imbalance than men in a healthy population [46] and in current/former smokers [42].

With regard to systemic lipid peroxidation, the multivariate analysis showed a significant correlation with gender but also with 6-MWD (% predicted). A possible explanation for the relationship between oxidative stress and the 6-MWD is that this submaximal test reflects the daily living conditions of COPD patients, unlike the maximal incremental test, for example, [47]. In literature, it is well admitted that high systemic lipid peroxidation levels induce cell dysfunction, which may lead to a decrease in muscle function contributing to exercise limitation, without evidence of differences between COPD men and women [2, 5, 8]. In our study, when COPD men and women were separated, we found significant negative correlation between systemic lipid peroxidation and 6-MWD (% predicted) only in COPD women, supporting the hypothesis of clinical and physiological differences between men and women in COPD advanced by De Torres et al. [18]. The present data therefore argue in favor of future supplementation with vitamins and trace elements to optimize the benefits of general nutritional support on muscle function/exercise capacity observed in COPD patients [48] by reducing deleterious OS as lipid peroxidation. Thus, we also suspect that COPD patients will show different positive responses to antioxidant supplements, validating specific phenotypes of patients with COPD [49].

In conclusion, our study showed for the first time that 89% of COPD patients had a systemic antioxidant imbalance, with more important deficits in vitamins and trace elements contributing to deleterious OS in 69% of them. Specific systemic OS profiles were defined in COPD patients and characterized by an increase in systemic copper and/or imbalance of vitamin C, GSH, and GPx associated with particularly high level of lipid peroxidation. In addition, from a clinical point of view, systemic lipid peroxidation level was higher in COPD women and was associated with a decrease in exercise capacity suggesting that different underlying mechanisms lead to muscle impairment. Future research is therefore needed to investigate more thoroughly the responses to targeted antioxidant supplementation in COPD patients.

## Figures and Tables

**Table 1:** Anthropometric and clinical characteristics of 54 COPD patients.

	COPD patients (*n* = 54)

Age (years)	60 ± 7
Sex ratio (W/M)	27/27
BMI (kg/m^2^)	24.7 ± 4.1
Fat-free mass index (kg/m^2^)	17.3 ± 2.3
Muscle mass index (kg/m^2^)	7.9 ± 1.5
FEV_1_ (% pred.)	54 ± 21
FEV_1_/FVC ratio	53 ± 14
GOLD stages I/II/III/IV (*n*)	6/23/20/5
BODE score [18]	2.1 ± 1.5
Breathlessness, MRC score	1.5 ± 1.1
Tobacco consumption (packs/year)	44 ± 24
Physical activity level, Voorips score [21]	4.4 ± 3.2
6-MWD (m)	518 ± 90
6-MWD (% pred.)	81 ± 14
W_sl_ (% pred.)	50 ± 19
VO_2_sl (% pred.)	60 ± 16
QMVC (Nm)	102 ± 37
QMVC (% pred.)	75 ± 19
Qend (s)	368 ± 152

Results are expressed in mean ± SD. COPD = chronic obstructive pulmonary disease; W = women/M = men; MRC = Medical Research Council; BODE index = body mass index, airway obstruction, dyspnoea, and exercise capacity index (6-MWD); BMI = body mass index (kg/m²); FEV_1_ (% pred.) = forced expiratory volume in 1 second; W_sl_ (% pred.) = symptom-limited power output; VO_2_sl (% pred.) = symptom-limited oxygen uptake; 6-MWD (m) = 6-minute walking distance; QMVC (Nm) = quadriceps maximal voluntary contraction expressed in Newtons; Qend (s) = quadriceps endurance time expressed in seconds.

**Table 2:** Systemic oxidative stress markers of 54 patients with COPD.

	Plasma levels of OS markers mean ± SD	COPD patients with deficits *N* (%)	Reference values (LLR–ULR)
Vitamin C (*µ*g/mL)	10.5 ± 4.2	14 (26%)	M: 6.21–15.18 F: 8.60–18.83
Vitamin E (mg/L)	14.4 ± 3.5	0 (0%)	8.00–15.00
Vitamin C/vitamin E	0.74 ± 0.30	17 (31%)	0.59–1.19
Selenium (*µ*g/mL)	89.5 ± 14.0	27 (66%)	94–130
Copper (mg/mL)	1.1 ± 0.2	3 (6%)	M: 0.70–1.40 F: 0.80–1.55
Zinc (mg/mL)	0.79 ± 0.13	15 (28%)	0.70–1.20
Copper/zinc ratio	1.42 ± 0.37	33 (61%)	1.14–1.29
GSH (*µ*mol/L)	944 ± 168	3 (6%)	717–1110
GSSG (*µ*mol/L)	5.40 ± 6.54	10 (19%)	0.96–10
GSH/GSSG	437 ± 320	11 (20%)	111–747
GPx (UI/gHb)	47.5 ± 12.1	3 (7%)	30–55
SOD (UI/gHb)	1310 ± 302	2 (6%)	785–1570
Lipid peroxidation (*µ*mol/L)	587 ± 256	37 (69%)	0–432
LDL ox (UI/L)	178 ± 275	16 (30%)	28–70

Results are expressed in mean ± SD. Percentage of COPD patients with selenium, GPx, and SOD deficits was established from a total of 41, 47, and 32 patients, respectively. COPD = chronic obstructive pulmonary disease; M = male/F = female; LLR and ULR = lower and upper limits of reference, respectively, obtained in a large cohort of healthy subjects; GSH = reduced glutathione; GSSG = oxidized glutathione; SOD = superoxide dismutase; GPx = peroxidase glutathione; LDL = oxidized low-density lipoprotein.

**Table 3:** Systemic OS levels in clusters of COPD patients.

	Cluster 1 *N* = 19	Cluster 2 *N* = 19	Cluster 3 *N* = 10	Cluster 4 *N* = 6	*p* interact.
Vitamin C (*µ*g/mL)	11.8 ± 4.0^*∗*^	8.8 ± 3.1^#^	14.0 ± 3.1^*∗*^	5.2 ± 2.7^#^	<0.001
Vitamin E (mg/L)	13.7 ± 2.9	14.5 ± 4.3	15.5 ± 3.6	14.4 ± 2.9	0.698
Vit. C/vit. E	0.86 ± 0.28^*∗*^	0.65 ± 0.26^#^	0.91 ± 0.14^*∗*^	0.36 ± 0.16^#^	<0.001
Selenium (*µ*g/mL)	92.3 ± 14.3	87.1 ± 13.9	91.7 ± 13.0	87.1 ± 17.6	0.744
Copper (mg/mL)	0.95 ± 0.16^*∗*#^	1.06 ± 0.12^*∗*#^	1.33 ± 0.26	1.34 ± 0.19	<0.001
Zinc (mg/mL)	0.82 ± 0.13	0.77 ± 0.09	0.74 ± 0.07	0.87 ± 0.20	0.161
Copper/zinc ratio	1.19 ± 0.25^*∗*#^	1.38 ± 0.21^#^	1.81 ± 0.44	1.60 ± 0.2	<0.001
GSH (*µ*mol/L)	945 ± 174	975 ± 150	978 ± 166	788 ± 153	0.096
GSSG (*µ*mol/L)	1.66 ± 0.97	11.84 ± 7.50^*∗*#§^	2.84 ± 0.80	1.15 ± 0.23	<0.001
GSH/GSSG	694 ± 279^#^	135 ± 113^*∗*#§^	363 ± 99^*∗*^	712 ± 213^#^	<0.001
GPx (UI/gHb)	47.8 ± 12.5	48.2 ± 13.9	51.1 ± 8.4	38.5 ± 7.9	0.265
SOD (UI/gHb)	1247 ± 260	1366 ± 332	1466 ± 311	1097 ± 222	0.219
Lipid peroxidation (*µ*mol/L)	402 ± 158^*∗*#^	570 ± 179^*∗*^	777 ± 253	904 ± 214	<0.001
LDL ox (UI/L)	172 ± 219	71 ± 57	98 ± 70	94 ± 99	0.188

Results are expressed in mean ± SD. ^*∗*^ *p* < 0.05 versus cluster 4; ^#^ *p* < 0.05 versus cluster 3; ^§^ *p* < 0.05 versus cluster 1. W = women; M = men; GSH = reduced glutathione; GSSG = oxidized glutathione; SOD = superoxide dismutase; GPx = peroxidase glutathione; LDL = oxidized low-density lipoproteins.

**Table 4:** Clinical, functional, and muscle characteristics in clusters of COPD patients.

	Cluster 1 *N* = 19	Cluster 2 *N* = 19	Cluster 3 *N* = 10	Cluster 4 *N* = 6	*p* interact.
Sex ratio (W/M)	8/11	5/14	8/2^*∗*#^	6/0^*∗*#^	<0.01

Age (years)	59 ± 7	63 ± 7	61 ± 8	57 ± 4	0.23
BMI (kg/m^2^)	24.0 ± 4.8	25.3 ± 2.8	25.4 ± 4.4	24.1 ± 4.8	0.71
Fat-free mass index (kg/m^2^)	17.4 ± 2.4	17.9 ± 2.2	16.6 ± 2.3	15.5 ± 1.7	0.14
Muscle mass index (kg/m^2^)	8.0 ± 1.6	8.5 ± 1.4	7.0 ± 1.5^#^	6.6 ± 0.6^#^	0.02
FEV_1_ (% pred.)	58 ± 24	50 ± 20	51 ± 13	61 ± 26	0.74
BODE score [18]	2.1 ± 1.3	1.6 ± 1.6	3.0 ± 1.4	1.8 ± 1.5	0.29
Breathlessness, MRC score	1.5 ± 1.3	1.5 ± 1.0	1.5 ± 1.3	1.3 ± 0.8	0.97
Tobacco consumption (packs/years)	43 ± 21	46 ± 26	34 ± 20	56 ± 27	0.31
Physical activity level, Voorips score [21]	4.6 ± 3.1	5.3 ± 4.1	4.1 ± 2.7	2.5 ± 1.1	0.69
6-MWD (m)	557 ± 87	516 ± 104	469 ± 35	481 ± 67	0.07
6-MWD (% pred.)	85 ± 14	79 ± 16	77 ± 10	79 ± 13	0.40
W_sl_ (% pred.)	53 ± 19	46 ± 16	49 ± 21	57 ± 24	0.53
VO_2_sl (% pred.)	62 ± 17	57 ± 16	64 ± 17	63 ± 19	0.68
QMVC (Nm)	98 ± 29	119 ± 45	90 ± 33	77 ± 16	0.11
QMVC (% pred.)	73 ± 14	81 ± 24	71 ± 16	68 ± 14	0.53
Qend (s)	397 ± 170	327 ± 105	337 ± 92	458 ± 253	0.70

Results are expressed in mean ± SD. ^*∗*^ *p* < 0.05 versus cluster 1; ^#^ *p* < 0.05 versus cluster 2. COPD = chronic obstructive pulmonary disease; W = women/M = men; MRC = Medical Research Council; BODE index = body mass index, airway obstruction, dyspnoea, and exercise capacity index (6-MWD); BMI = body mass index (kg/m²); FEV_1_ (% pred.) = forced expiratory volume in 1 second; W_sl_ (% pred.) = symptom-limited power output; VO_2_sl (% pred.) = symptom-limited oxygen uptake; 6-MWD (m) = 6-minute walking distance; QMVC (Nm) = quadriceps maximal voluntary contraction expressed in Newtons; Qend (s) = quadriceps endurance time expressed in seconds.

**Table 5:** Parameters associated with systemic lipid peroxidation in 54 patients with COPD (multivariate analysis: ANCOVA).

	Df	Sum Sq	Mean Sq	*F* value	Pr (>*F*)
Gender	1	421026	421026	7.46	0.01^*∗*^
Age (years)	1	53879	53879	0.96	0.33
Tobacco consumption (packs/year)	1	13984	13984	0.25	0.62
Physical activity level, Voorips score	1	48971	48971	0.87	0.36
Muscle mass index (Kg/m^2^)	1	2134	2134	0.04	0.84
FEV_1_ (% pred.)	1	134549	134549	2.39	0.14
FEV_1_/FVC	1	17162	17162	0.31	0.59
6-MWD (% pred.)	1	291300	291300	5.18	0.03^*∗*^
Residuals	22	1239096	56323		

To check parameters associated with systemic lipid peroxidation, we performed covariance analysis (ANCOVA). ^*∗*^ *p* < 0.05 means that the parameter is statistically associated with systemic lipid peroxidation. COPD = chronic obstructive pulmonary disease; BMI = body mass index; FEV_1_ = forced expiratory volume in 1 second; FVC = forced vital capacity; 6-MWD = 6-minute walking distance; Df = degrees of freedom; Sq = square.
